# Clinical Urinology

**Published:** 1904-12

**Authors:** 


CLINICAL URINOLOGY. By ALFRED C. CROFTAN, Professor of Medicine, Chicago Post-Graduate Medical College and Hospital. Octavo, pp. 314. Illustrated. New York: William Wood & Company. 1904. (Price, $2.50 net.)

Sometimes a publisher is wise enough and sufficiently far-sighted to see the benefit of taking the middle of the road when the way is pointed out by a man with new ideas. This is what Wood & Company have done in the case of Croftan's book just issued.

There have been handbooks and laboratory guides and treatises and what-not on the analysis of urine; there have been highly polished and sometimes well-written books on the diseases which produce urinary disorders. This book is neither one nor the other, and in a preface which is both modest and brief, and hence refreshing, Croftan makes this point clear, and establishes his book on "the borderline that lies between the laboratory and the clinic."

An author may be a pioneer in a certain field, yet if he lack the boldness which marks the true leader, if he has not the courage to disdain hide-bound tradition and perfunctory precedent, he but faintly marks a foot path for some more courageous spirit to follow and blaze the open trail.

It was more than an ordinary task, the writing of this book, and it may be that this fact will not be immediately appreciated; but eventually it will be looked upon as the first guiding light into a new field. The present day knowledge of clinical urinology and urinary pathology is sadly incomplete. Far less advancement has been made in this than in most other branches of medicine; but that a turn in the right direction has been taken and that rapid progress is being made is evidenced by the completeness, the novelty and the refreshing originality of Croftan's book, which is not alone a well-written work on the subject, but is just the bold, aggressive idol-smasher one might expect from a Chicago author; one who knows how to express himself clearly and possesses Lydston's Chicagoesque knack of carelessly tossing aside hoary and time-riddled fads and fancies which have been tolerated too long, and tolerated chiefly because we humans have been brought up to respect age in whatever form we meet it.

The chemic ingredients of the urine are grouped according to their chemic relationship, and the factors determining their excretion in normal and pathologic conditions are discussed broadly and without so much as "by-your-leave" to any tradition or former arrangement.

The author evidently, and very properly it seems, leans to the belief that too little attention has been paid in the past to the inorganic constituents of the urine, for he devotes considerable space to what he terms "these cinders of metabolism."

The chapter on the determination of renal function is particularly clear and of immense value, as is the chapter on sediments in which particular stress is laid on their origin.

Finally, it will come as something akin to shock to many to miss Ehrlich's diaze-reaction, together with several other things in the way of urinary frills, such as the fluctuating ratio between urea and uric acid; the phosphates and urea and total nitrogen. All these and several lesser lights emanating from French authors have stared us out of countenance from nearly every textbook too long. Urologists who are abreast of the times have long since discarded these fancies as being unimportant, unreliable and wholly lacking a basis of scientific fact. Croftan, however, is the first writer to publicly ignore these paste jewels of urinology. N. W. W.

